# Mixing Performance of a Planar Asymmetric Contraction-and-Expansion Micromixer

**DOI:** 10.3390/mi13091386

**Published:** 2022-08-25

**Authors:** Daigo Natsuhara, Ryogo Saito, Shunya Okamoto, Moeto Nagai, Takayuki Shibata

**Affiliations:** Department of Mechanical Engineering, Toyohashi University of Technology, Toyohashi 441-8580, Japan

**Keywords:** passive micromixer, planar asymmetric contraction-and-expansion micromixer, microfluidic device, lab-on-a-chip

## Abstract

Micromixers are one of the critical components in microfluidic devices. They significantly affect the efficiency and sensitivity of microfluidics-based lab-on-a-chip systems. This study introduces an efficient micromixer with a simple geometrical feature that enables easy incorporation in a microchannel network without compromising the original design of microfluidic devices. The study proposes a newly designed planar passive micromixer, termed a planar asymmetric contraction-and-expansion (P-ACE) micromixer, with asymmetric vertical obstacle structures. Numerical simulation and experimental investigation revealed that the optimally designed P-ACE micromixer exhibited a high mixing efficiency of 80% or more within a microchannel length of 10 mm over a wide range of Reynolds numbers (0.13 ≤ *Re* ≤ 13), eventually attaining approximately 90% mixing efficiency within a 20 mm microchannel length. The highly asymmetric geometric features of the P-ACE micromixers enhance mixing because of their synergistic effects. The flow velocities and directions of the two fluids change differently while alternately crossing the longitudinal centerline of the microchannel, with the obstacle structures asymmetrically arranged on both sidewalls of the rectangular microchannel. This flow behavior increases the interfacial contact area between the two fluids, thus promoting effective mixing in the P-ACE micromixer. Further, the pressure drops in the P-ACE micromixers were experimentally investigated and compared with those in a serpentine micromixer with a perfectly symmetric mixing unit.

## 1. Introduction

Lab-on-a-chip (LOC) technology affords a wide range of potential applications in various fields, from chemistry, biology, and medicine [1,2,3,4,5,6,7] to environmental health and food safety diagnostics [8,9,10]. Compared with conventional techniques, LOC platforms offer outstanding capabilities that involve inherently less consumption of samples and reagents, shorter reaction and analysis time, higher resolution and sensitivity, high-throughput parallelized and fully automated sample processing, less expensive instrumentation, and operational simplicity. This leads to higher cost-effectiveness while maintaining or even enhancing the functionalities. However, to offer such unique features, LOC devices should incorporate multiple microfluidic components such as pumps, valves, mixers, filters, separators, dispensers, reactors, and other elements necessary to facilitate precise fluid handling. Furthermore, additional sensing elements have to be combined on the same device to enable fully automated stand-alone LOC systems.

Fluid flow is dominated by laminar flow in microchannels owing to their extremely low Reynolds numbers, posing difficulty in the mixing of fluids. Therefore, micromixers are among the crucial components that have a considerable impact on the efficiency and sensitivity of microfluidic devices. To date, numerous attempts have been undertaken to develop highly efficient micromixers that are broadly classified as active and passive micromixers [11,12,13,14,15,16]. Active micromixers exhibit a high mixing efficiency of over 90% within a short mixing length and/or a short mixing time over a wide range of Reynolds numbers (0.01 < *Re* < 100). However, they require external energy sources such as pressure, electric, magnetic, thermal, and acoustic fields [11,12,13,14] to enhance the mixing process by stirring or agitating the fluids. Thus, the systems and equipment involved are complicated and costly. In contrast, passive micromixers do not require external energy sources except for a pumping system to deliver fluids into microfluidic devices [15,16]. Thus, passive micromixers are more appropriate for enabling on-site diagnostic testing or point-of-care testing platforms. However, compared with active micromixers, passive micromixers generally lack mixing ability, and the mixing process relies mainly on molecular diffusion or chaotic advection.

The mixing efficiencies of representative passive micromixers are summarized in Table 1. In passive micromixers, T-shaped and Y-shaped channel micromixers have the simplest geometrical configurations and allow the easiest fabrication process. However, efficient mixing performance can be achieved at relatively high Reynolds numbers (*Re* > 400) [17,18]. Hossain et al. [19] conducted a numerical investigation of the mixing performance of serpentine micromixers with three different microchannel geometries: curved, square-wave, and zigzag. The results showed that the square-wave microchannel exhibited better mixing efficiency than the curved and zigzag microchannels over a wide range of Reynolds numbers (0.267 < *Re* < 267). However, a sufficiently high mixing efficiency (>90%) was achieved at a high Reynolds number (*Re* = 267) for all three microchannel geometries. Similar results for the three microchannel geometries have been reported by Chen et al. [20]. Both experimental and numerical simulation results showed that the mixing efficiency decreased with an increase in *Re* from 0.1 to 1. The mixing efficiency subsequently increased with an increase in *Re* from 1 to 100, attaining a mixing efficiency of 95% at *Re* = 100. Tsai and Lin [21] experimentally investigated zigzag serpentine micromixers with six different zigzag angles ranging from 0° (a straight channel) to 75° (a zigzag channel with the sharpest turning corner) for Reynolds numbers between 0.309 and 309. They reported a clear transition between diffusion-based and advection-based mixing at *Re* ≈ 30; thus, a mixing efficiency of approximately 90% was achieved at *Re* > 100. Wang et al. [22] designed serpentine micromixers with six different types of ellipse-curved microchannels. The experimental and numerical simulation results (*Re* = 0.1–100) suggested that the mixing efficiency was above 90% at *Re* = 0.1 and *Re* > 80.

Hong et al. [23] proposed a novel in-plane passive micromixer with a two-dimensional (2D) modified Tesla structure. The numerical simulation and experimental results (0.1 < *Re* < 10) revealed that the mixing efficiencies of the 2D Tesla micromixer were approximately 70% and 90% at *Re* = 0.1–1 and *Re* = 5–10, respectively. Hossain et al. [24] performed a numerical optimization study on the geometrical dimensions of a modified Tesla micromixer in the Reynolds number range of 0.05 to 40. The numerical simulations showed that a high mixing efficiency (>80%) was achieved at *Re* = 0.05 and *Re* = 40, whereas the mixing efficiency (<40%) reached a minimum at *Re* = 2. These results suggest that molecular diffusion remains the dominant phenomenon for mixing in a 2D Tesla micromixer. Yang et al. [25] demonstrated a three-dimensional (3D) Tesla micromixer that exhibited excellent mixing efficiency (>95% within an axial distance of 5.5 mm) over the Reynolds number ranging from 0.1 to 100. To date, various types of passive micromixers with complex 3D geometrical structures have been proposed to improve mixing performance. Examples of these include C-shape [26] and L-shape [27] serpentine micromixers and H-shape [28] and H-C shape [29] split-and-recombine (SAR) micromixers. Le The et al. [30] proposed a shifted-trapezoidal-blades micromixer in which a high mixing efficiency (>80%) was achieved over a wide range of Reynolds numbers (*Re* = 0.5–100) owing to the combination of several mixing principles, including vortices, transversal flows, and chaotic advection. The highest mixing efficiency was 95% at *Re* = 40.

In another type of passive micromixer, obstacles are periodically embedded in a microchannel to generate chaotic advection. Stroock et al. [31] first demonstrated obstacle-based micromixers (the so-called chaotic micromixer), in which periodic obliquely oriented ridges (referred to as a slanted-groove micromixer, SGM) or staggered herringbone structures (referred to as a staggered-herringbone mixer, SHM) were fabricated on the bottom surface of a microchannel to generate a transverse component in the flow. The experimental results showed that the mixing efficiencies of the SHM exceeded 90% within a 30 mm channel length over a wide range of Reynolds numbers (*Re* = 0.2–90). Ianovska et al. [32] experimentally identified that the SHM exhibited better mixing efficiency than the SGM, resulting in a high mixing efficiency of over 90% within a 10 mm channel length for *Re* ranging from 0.3 to 90. Kim et al. [33] presented a new chaotic passive micromixer called a barrier-embedded micromixer (BEM). In the BEM, periodically located rectangular obstacles parallel to the flow direction were embedded on the top surface of the SGM configuration to generate more complex chaotic advection. The experimental results demonstrated that the mixing efficiency of the BEM reached over 80% within a 20 mm channel length for *Re* = 0.2–2.

As described above, the SHM micromixer is one of the most efficient chaotic advection-based micromixers. However, it requires a complicated fabrication process, that is, a two-step photolithography and alignment processes. As another type of obstacle-based micromixer designed with simple planar structures, a passive micromixer with triangle baffles fabricated via only a single-step photolithography process was proposed by Wang et al. [34]. The experimental and numerical simulation results revealed that the highest mixing efficiency of 91.2% at a channel length of 6.4 mm could be achieved at *Re* = 0.1. However, the mixing efficiency decreased with increasing the Reynolds number. The mixing efficiencies were 85.5% at *Re* = 1, 79.5% at *Re* = 10, and 57.9% at *Re* = 500. Fang et al. [35] proposed a passive micromixer with simple geometric features in which parallelogram obstacle structures inclined with respect to the flow direction were designed alternately on both sidewalls of a straight microchannel. The numerical simulation results indicated that the mixing efficiency reached 79.4% at a channel length of 1.4 mm at *Re* = 0.29. Li et al. [36] numerically and experimentally investigated the performance of a modified planar asymmetric split-and-recombine (P-SAR) micromixer at *Re* = 1–100. The mixing performance was enhanced as a result of a synergistic combination of unbalanced inertial collisions, multi-directional vortices, and converging–diverging flow. The highest mixing efficiency of 86% was achieved at *Re* = 80, whereas the lowest mixing efficiency (<20%) was achieved at *Re* = 10. Moreover, Scherr et al. [37] proposed a passive planar micromixer with a logarithmic spiral microchannel. The experimental and numerical simulation results showed that the mixing efficiency initially decreased from 80% or more at *Re* = 1 to a minimum mixing efficiency of 53% at *Re* = 15. The mixing efficiency subsequently increased with an increase in *Re*, reaching a maximum value of 86% at *Re* = 67. To date, several types of planar passive micromixer geometries have been studied numerically and experimentally. These include an accordion-shaped micromixer [38], a micromixer with circular and square chambers [39], a micromixer with obstacle-laden fish-shaped and spiral-shaped microchannels [40], and a hybrid micromixer with planar mixing units [41].

In summary, active micromixers and 3D passive micromixers demonstrate a high mixing efficiency over a wide range of the Reynolds number. However, they typically require complex fabrication and assembly processes. Thus, not only do they pose difficulty in integration into microfluidic devices, but they are also less cost-effective for use in LOC applications. Moreover, planar passive micromixers have many advantages, such as ease of fabrication and high integrability as fluidic components of microfluidic devices. However, their highly efficient mixing performance is typically limited to either a relatively low Reynolds number (*Re* < 0.1, dominated by molecular diffusion) or a relatively high Reynolds number (*Re* > 50, dominated by chaotic advection). Therefore, there is scope for further improvement in the mixing performance of planar passive micromixers over a wide range of the Reynolds number. Our previous studies [42,43,44,45] developed a versatile microfluidic device for the multiplexed detection of targeted nucleic acids based on the loop-mediated isothermal amplification method. In this study, to simplify the operating procedure for our targeted applications, we propose a simple and efficient planar passive micromixer with asymmetric vertical obstacle structures for easy integration into microfluidic devices. Experimental and numerical investigations of the mixing efficiency and pressure drop were conducted in the *Re* range of 0.13 to 13 for the micromixer design optimization.

**Table 1:** Mixing efficiencies of representative passive micromixers.

Dimension	Categories	Characteristics	*Re*	Mixing Efficiency (Max.)	Mixing Efficiency (Min.)	Ref.
2D	Lamination	T-shaped	100–1400	~100% ^f^ (*Re* = 400–500)		[17] ^c^
		T-shaped	0.5–550	~98% ^f^ (*Re* > 300)	10% (*Re* = 3–35)	[18] ^a^
		Modified 2D Tesla	0.1–10	~ 90% ^f^ (*Re* = 5–10)		[23] ^c^
		Modified 2D Tesla	0.05–40	80–90% ^d^ (*Re* = 0.05, *Re* = 40)	< 40% (*Re* = 2)	[24] ^b^
		Asymmetric split-and-recombine (P-SAR)	1–100	86% ^d^ (*Re* = 80)	< 20% (*Re* = 10)	[36] ^c^
	Serpentine	Curved, square-wave, and zigzag	0.267–267	90% ^d^ (*Re* = 267)	10% (*Re* = 5–15)	[19] ^b^
		Curved, square-wave, and zigzag	0.1–100	95% ^e^ (*Re* = 100)	40–50% (*Re* = 1)	[20] ^c^
		Zigzag	0.309–309	90% ^e^ (*Re* > 100)	40% (*Re* = 30)	[21] ^a^
		Ellipse-curved	0.1–100	90% ^d^ (*Re* = 0.1, *Re* > 80)	25% (*Re* = 1–10)	[22] ^c^
		Logarithmic spiral	1–70	80–86% ^f^ (*Re* = 1, *Re* = 67)	53% (*Re* = 15)	[37] ^c^
	Obstacle	Triangle baffles	0.1–500	86% ^d^ (*Re* = 0.1)	58% (*Re* = 500)	[34] ^c^
		Parallelogram	0.29	80% ^d^ (*Re* = 0.29)		[35] ^c^
3D	Lamination	3D Tesla	0.1–100	95% ^e^ (*Re* = 0.1–100)		[25] ^c^
		Sifted trapezoidal blades	0.5–100	80–95% ^e^ (*Re* = 0.5–100)		[30] ^c^
	Obstacle	Staggered herringbone (SHM)	0.2–90	90% ^d^ (*Re* = 0.2–90)		[31] ^a^
		Staggered herringbone (SHM)	0.3–90	90% ^d^ (*Re* = 0.3–90)		[32] ^a^
		Barrier-embedded (BEM)	0.2–2	80–90% ^f^ (*Re* = 0.2–2)		[33] ^a^

^a^ Research including only experimental results. ^b^ Research including only numerical simulation results. ^c^ Research including experimental and numerical simulation results. ^d^ Mixing efficiency (ME) is defined as follows: $\mathrm{ME}=1-\frac{\sqrt{\frac{1}{N}\sum_{i=1}^{N}{\left(c_{i}-\bar{c}\right)}^{2}}}{SD_{0}}$, where *N* is the total number of sampling points across the width of the microchannel, and $c_{i}$ and $\bar{c}$ are the point concentration (or pixel intensity) and the mean concentration (or pixel intensity), respectively. $SD_{0}$ is the maximum standard deviation in the non-mixing region at the inlet of the microchannel. ^e^ Mixing efficiency (ME) is defined as follows: $\mathrm{ME}=1-\frac{\sqrt{\frac{1}{N}\sum_{i=1}^{N}{\left(c_{i}-\bar{c}\right)}^{2}}}{\bar{c}}$. ^f^ Mixing efficiency is defined in different ways.

## 2. Materials and Methods

### 2.1. Computational Analysis

**Figure 1:**
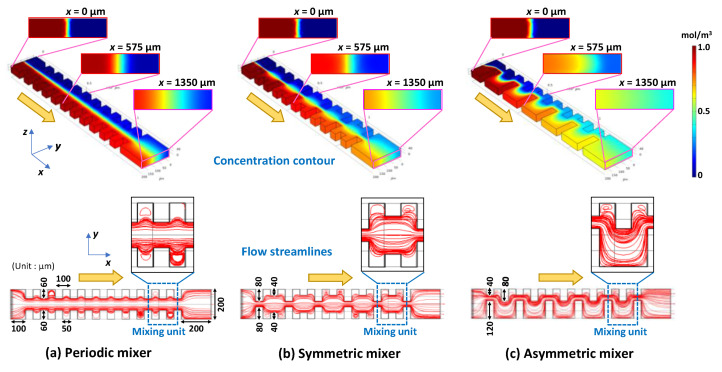
Simulation results showing the flow streamlines and concentration contours of two different fluids (with a concentration of 0 and 1 mol/m^3^) in three types of planar micromixers with different types of simple vertical obstacle structures at a flow velocity of 8.3 × 10^–3^ m/s each (equivalent to a total flow rate of 10 µL/min and *Re* = 1.3). (**a**) Periodic mixer with periodic rectangular obstacle structures; (**b**) symmetric mixer with two types of symmetric obstacle structures alternately arranged with two different gap distances; (**c**) asymmetric mixer with two types of asymmetric obstacle structures alternately arranged with two different gap distances.

Our previous study [42] demonstrated that a simple configuration of periodic structures with oblique ridges fabricated in a straight microchannel is most suitable for a passive mixer (known as a chaotic mixer [31]). A mixing efficiency of approximately 90% within a microchannel length of 10 mm could be attained. However, the fabrication process of this type of mixer requires the utilization of complicated two-step photolithography and alignment processes. Therefore, this study proposes a newly designed, simple, and efficient planar passive micromixer with asymmetric vertical obstacle structures that can be fabricated using a single-step photolithography process. The micromixer developed can be integrated into polydimethylsiloxane (PDMS)-based microfluidic devices employed for rapid and easy-to-use multiplexed genetic diagnostics [42,43,44,45]. A numerical comparison of the mixing efficiencies of the three types of planar mixers with simple vertical obstacle structures (Figure 1) was performed via the finite element method (FEM) using commercially available software (COMSOL Multiphysics version 5.4, COMSOL AB, Stockholm, Sweden). The geometrical configurations and dimensions of each mixer with three different types of vertical obstacle structures (50 µm in width, 50 µm in height, 100 µm in pitch, and 40–120 µm in length) embedded on both sidewalls of a rectangular microchannel (200 µm in width, 50 µm in height, and 1.35 mm in length) are shown in Figure 1. The mixing phenomena were analyzed under steady-state conditions by coupling the single-phase flow and transport of the diluted species modules of COMSOL. The fluid was assumed to be incompressible, and a no-slip boundary condition was imposed on the surface of the microchannel wall. The three-dimensional FEM models (approximately 180,000 elements) for each micromixer were simplified by reducing the number of periodic obstacle structures and their sets (defined as the mixing unit in Figure 1) to reduce the computational resources and time required for the analysis. Assuming the fluid to be water, the density and dynamic viscosity of the two fluids were set to 1.0 × 10^3^ kg/m^3^ and 1.0 × 10^–3^ kg/m·s, respectively. To consider the mixing of two fluids, blue-colored fluid (with a concentration of 0 mol/m^3^) and red-colored fluid (with a concentration of 1 mol/m^3^) were introduced into a microchannel at a flow velocity of 8.3 × 10^–3^ m/s each, equivalent to 5 µL/min for each fluid. Thus, the total flow rate and Reynolds number (*Re*) were 10 µL/min and 1.3, respectively, in the rectangular microchannel without obstacle structures. The pressure at the outflow boundary was set to zero. A diffusion coefficient of 4.25 × 10^–10^ m^2^/s at 25 °C for fluorescein [46] was used in the numerical simulations. The mixing efficiency (ME) was calculated using ME (%) = (1 − *SD_x_*/*SD*_0_) × 100 [13,42], where *SD*_0_ and *SD_x_* were the standard deviations of the concentration estimated at the inlet and outlet positions of the microchannel in the numerical simulation results, respectively. The definition of the mixing efficiency corresponds to d in the footnote of Table 1. It should be noted that, in the aforementioned experimental results, *SD*_0_ and *SD_x_* were estimated at the initial position *x* = 0 (which was set 270 µm away from the entrance of the first mixing unit) and at an arbitrary position *x* (mm) (defined as the microchannel length from the initial position) in the longitudinal direction of the microchannel, respectively.

### 2.2. Fabrication Process and Experimental Investigation

The micromixers with obstacle structures embedded on both sidewalls of a Y-shaped rectangular microchannel (200 µm in width and 50 µm in height) were fabricated through a soft-lithography process using a thick negative photoresist (SU-8 3050, MicroChem Corp., Newton, MA, USA) as a mold. The SU-8 master mold was replicated in PDMS (Silpot 184, Dow Corning Toray Co., Ltd., Tokyo, Japan) after curing at 80 °C for 40 min. After punching holes for the inlet and outlet ports (1.0 mm in diameter), the microchannels were sealed with a glass substrate (S9213, Matsunami Glass Ind., Ltd., Osaka, Japan) using a silicone-based adhesive double-sided tape (No. 5303W, Nitto Denko Corp., Osaka, Japan). The water contact angles of PDMS and the adhesive tape were 108° and 102°, respectively.

To investigate the mixing behavior in the fabricated micromixers, fluorescence imaging was performed using an inverted microscope (TE2000-U, Nikon, Tokyo, Japan). Pure water and fluorescein-dyed water (0.1 mol/m^3^) were introduced into the microchannel from individual inlet ports at the same flow rates ranging from 0.5 to 50 µL/min with two syringe pumps (YSP-201, YMC Co., Ltd., Kyoto, Japan) (equivalent to total flow rates ranging from 1 to 100 µL/min and *Re* range of 0.13 to 13 in the microchannel). The experimental investigation of the pressure drop used two types of pressure-driven micropumps with different maximum pressure capabilities (Flow EZ^TM^ 345 mbar and 7000 mbar used in the flow rate ranges of 1–10 µL/min and 20–100 µL/min, respectively; Fluigent SA, Le Kremlin-Bicêtre, France) equipped with a flow sensor (Flow Unit, Fluigent SA). The micropumps controlled the desired flow rate of water (colored with blue food color, 0.1% *w*/*v*) introduced into a microchannel. Additionally, they facilitated the measurement of the overall pressure drop required for liquid delivery from one of the two inlet ports (the other was not opened in the PDMS devices) to an outlet port.

## 3. Results and Discussion

### 3.1. Computational Analysis of Mixing Phenomena in a Microchannel

As shown in Figure 1a, the first micromixer consisted of simple periodic geometric features (hereinafter referred to as periodic mixers). The rectangular obstacle structures (50 µm in width and 60 µm in length) that face each other from both sidewalls of the microchannel were arranged regularly, resulting in a narrow gap of 80 µm. The flow streamlines exhibited an almost laminar flow pattern. In addition, the concentration contour shows that the interface between the two fluids was visible owing to laminar flow, thus resulting in a low mixing efficiency of 47% at *Re* = 1.3 at a microchannel length of 1.35 mm. The streamlines of the two liquids were asymmetric because the 3D mesh model generated automatically in the FEM simulations was not perfectly symmetric with respect to the longitudinal centerline of the microchannel. The second micromixer (hereinafter referred to as symmetric mixer, as shown in Figure 1b) was expected to improve the mixing efficiency based on the change in the flow velocity owing to the two types of alternately arranged obstacle structures with different gap distances of 40 µm and 120 µm. The mean value of the gap distances was designed to be the same as that of the periodic mixer. The flow streamlines were spread over the cross-sectional area of the microchannel, and the interface between the two fluids repeatedly contracted and expanded, as indicated by the concentration contour. As a result, the mixing efficiency increased by 67%. The third micromixer (hereinafter referred to as the asymmetric mixer, as shown in Figure 1c) featured the same gap distances (40 µm and 120 µm) as the symmetric mixer, whereas the center position of the gaps was alternately changed for each obstacle structure; that is, it was designed to be asymmetric with respect to the longitudinal centerline of the microchannel. The geometric features could promote mixing by not only changing the flow velocities of the two fluids but also by intentionally changing the flow directions of the two fluids along the traverse direction of the microchannel. The flow streamlines and concentration contour demonstrated that the asymmetrical flow induced by the asymmetric obstacle structures markedly enhanced the mixing, thus significantly improving the mixing efficiency up to 84%.

### 3.2. Experimental Investigation of Mixing Phenomena in a Microchannel

**Figure 2:**
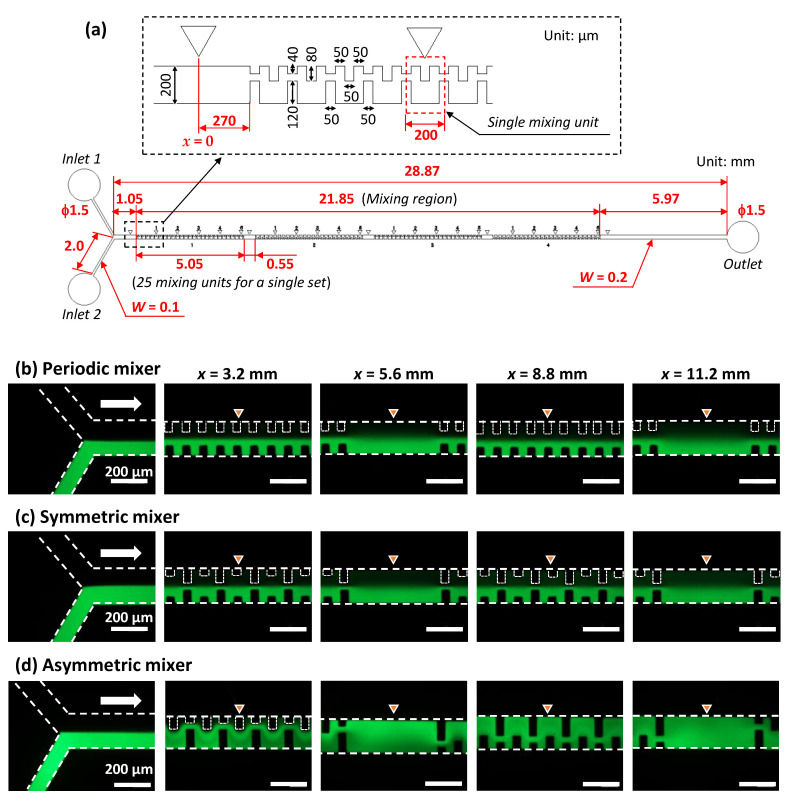
Experimental investigation of mixing behaviors. (**a**) Detailed design of the asymmetric mixer employed in the experiments. Experimental results showing fluorescence microscopy images of the mixing process in the (**b**) periodic mixer, (**c**) symmetric mixer, and (**d**) asymmetric mixer. Pure water and fluorescein-dyed water (0.1 mol/m^3^) were introduced at the same flow rate of 5 µL/min (a total flow rate of 10 µL/min and *Re* = 1.3).

To verify the simulation results, we experimentally investigated the mixing efficiency of the asymmetric mixer and compared it with those of the periodic mixer and symmetric mixer. The detailed design of the asymmetric mixer employed in the experiments is illustrated in Figure 2a, which features a Y-shaped configuration consisting of two inlet ports and one outlet port and a rectangular straight microchannel with a length of 28.87 mm. The mixing regime consisted of four sets of 25 pairs of the mixing units (defined as the mixing unit in Figure 2a) that were fabricated inside a PDMS microchannel (200 µm in width and 54 µm in height). The 100 mixing units occupied 21.85 mm of the microchannel length, including a 0.55 mm spacing between each set. In addition, the orientation of each set (25 mixing units for a single set) of the asymmetric obstacle structures was reversed with respect to the flow direction in the microchannel. For comparison, the periodic mixer and symmetric mixer were also fabricated with similar configurations, but their geometric features were perfectly symmetric with respect to the longitudinal centerline of the microchannel.

Figure 2b–d shows the fluorescence microscopy images of the flow behavior of two different fluids in each micromixer within the first two sets of 25 pairs of the mixing units, that is, the periodic mixer, symmetric mixer, and asymmetric mixer. In the present experiments, the zero position (*x* = 0 as a non-mixing initial position) was set 270 µm away from the entrance of the first mixing unit, as shown in Figure 2a. The inverted triangle marks denoted in the photographs indicate the distance *x* (mm) from the zero position (*x* = 0). As predicted from the simulation results (Figure 1a), the flow profile in the periodic mixer (Figure 2b) exhibited unimpeded laminar flow, as if there were no obstacle structures on both sidewalls of the microchannel. In the case of the symmetric mixer (Figure 2c), the steady laminar flow profile was maintained, although the interface between the two fluids repeatedly contracted and expanded owing to the change in the flow velocity resulting from the alternate arrangement of two types of obstacle structures with a narrow and wide gap distance (40 µm and 120 µm, respectively). In contrast, the asymmetric mixer (Figure 2d) demonstrated a uniform intensity of green fluorescence across the microchannel width, even after passing through the first set of 25 mixing units (*x* = 5.6 mm).

**Figure 3:**
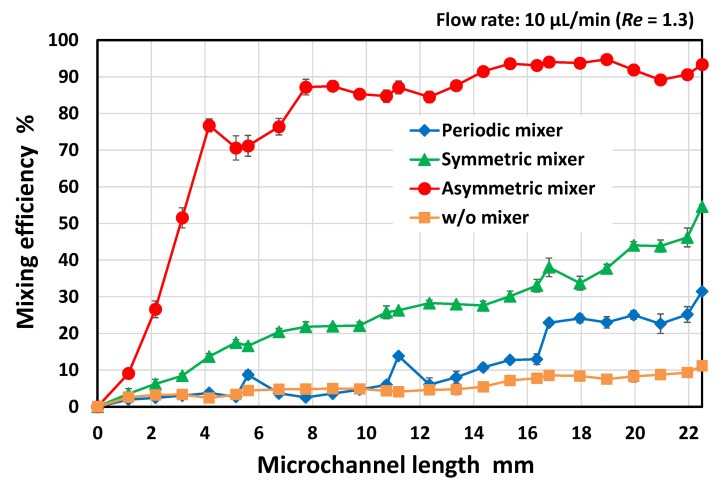
Experimental results showing the mixing efficiencies of the periodic mixer, symmetric mixer, and asymmetric mixer (as shown in Figure 2) as a function of the microchannel length. For comparison, the data obtained in a rectangular microchannel without obstacle structures is also plotted in the graph. Note that each point and error bar in the plot represents the mean values and standard deviations, respectively.

The resulting mixing efficiencies are plotted in Figure 3 as a function of the microchannel length (defined as an arbitrary position *x* (mm) from the initial position *x* = 0), where the vertical bars represent the standard deviation for each mixer in the three experiments. For comparison, the data obtained for a rectangular microchannel without obstacle structures are also plotted in the graph. To quantify the mixing efficiency, the standard deviation of the fluorescence intensities over the microchannel cross-section (approximately 50 µm × 200 µm region) was estimated using ImageJ software (version 1.53e, National Institutes of Health, Bethesda, MD, USA). The mixing efficiency in a rectangular microchannel without any mixer reached only 10% after the two fluids passed through the microchannel at a length of 20 mm. In addition, the periodic mixer and the symmetric mixer with symmetric geometric features with respect to the longitudinal centerline of the microchannel also exhibited a low mixing efficiency of approximately 25% and 45%, respectively, at a microchannel length of 20 mm. In contrast, the mixing efficiency of the asymmetric mixer was remarkably improved—80% or more at a microchannel length of 10 mm (after passing through the first two sets of 25 mixing units) and subsequently approaching 90% or more at a microchannel length of 15 mm (after passing through the third set of the mixing units). The results suggest that the asymmetric geometric features enhanced the mixing performance possibly because of the significant synergistic effect in which the flow velocities and flow directions of the two fluids toward the traverse direction of the microchannel were intentionally changed by the obstacle structures asymmetrically arranged with respect to the longitudinal centerline of the microchannel.

### 3.3. The Effect of the Number of Mixing Units

**Figure 4:**
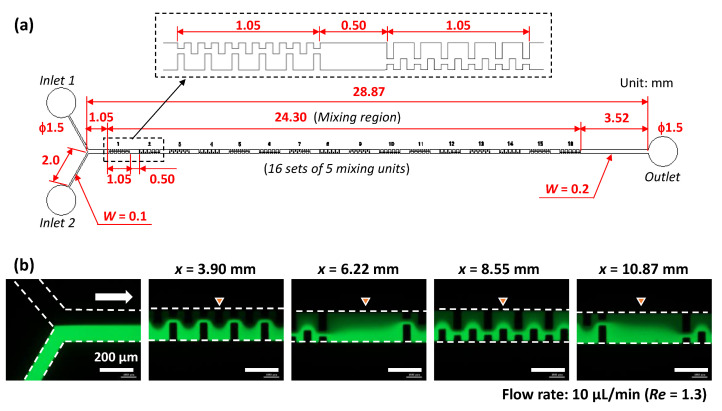
Experimental investigation of the influence of the number of mixing units for a single set on the mixing efficiency. (**a**) Detailed design of the asymmetric mixer with sixteen sets of five mixing units employed in the experiments. (**b**) Fluorescence microscopy images of the mixing process, in which pure water and fluorescein-dyed water (0.1 mol/m^3^) were introduced at the same flow rate of 5 µL/min (a total flow rate of 10 µL/min and *Re* = 1.3).

We experimentally investigated the effect of the number of mixing units arranged in the same orientation for a single set on the mixing efficiency. As shown in Figure 4a, the mixing regime consisted of sixteen sets of five mixing units (with the same geometric dimensions as the asymmetric mixer as shown in Figure 2d) fabricated in a microchannel. The 100 mixing units occupied 24.30 mm of the microchannel length, including a 0.50 mm spacing between each set. The orientation of each set (five mixing units for a single set) of asymmetric obstacle structures was reversed with respect to the flow direction in the microchannel. Figure 4b shows the fluorescence microscopy images of the flow behavior of pure water and fluorescein-dyed water (0.1 mol/m^3^) introduced into the microchannel (200 µm in width and 54 µm in height) at a flow rate of 5 µL/min each. Thus, the total flow rate was 10 µL/min and *Re* = 1.3. The flow maintained a steady laminar profile after passing through seven sets of five mixing units (*x* = 10.87 mm), although the interface between the two fluids gradually disappeared. In addition, there was no evidence that the flow positions of the two fluids alternatively changed across the width direction of the microchannel. This flow pattern was different from those generated in the asymmetric mixers consisting of four sets of 25 mixing units (Figure 2d).

**Figure 5:**
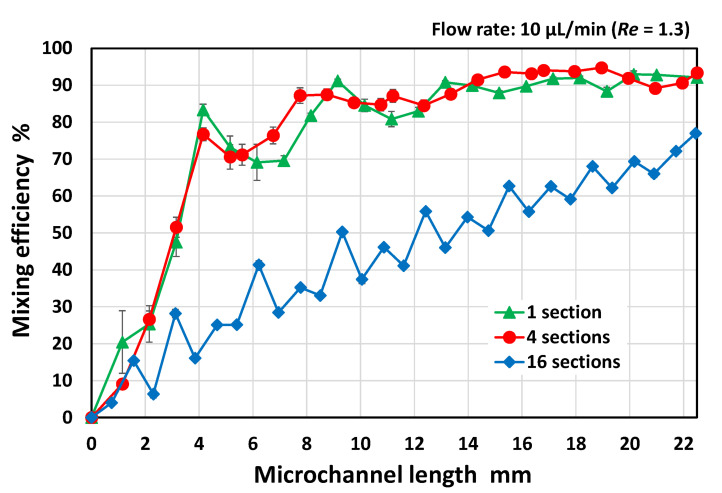
Experimental results showing the mixing efficiencies of three different types of asymmetric micromixers (M3) with different numbers of mixing units for a single set at a total flow rate of 10 µL/min (*Re* = 1.3). For comparison, the mixing efficiencies of the asymmetric mixers with four sets of twenty-five mixing units and a single set of one hundred mixing units are also plotted in the graph based on the data shown in Figure 3 and Section 3.4, respectively.

**Figure 6:**
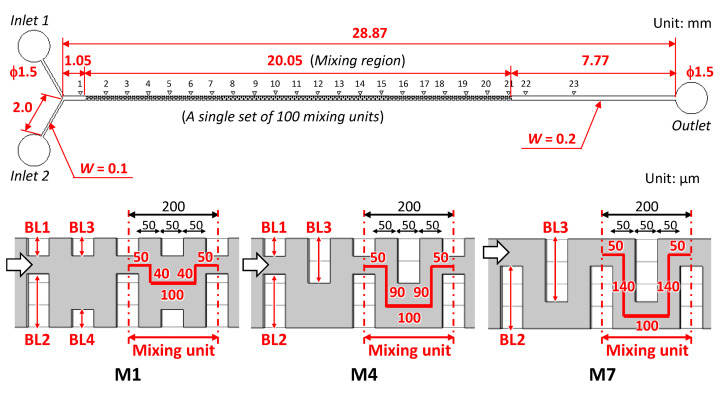
Detailed design of the asymmetric mixers consisting of a single set of 100 mixing units employed in the experiments to investigate the influence of the geometrical configurations and dimensions of the asymmetric mixers on the mixing efficiency.

As shown in Figure 5, efficient mixing did not occur in the mixer consisting of sixteen sets of five mixing units compared with the mixer consisting of four sets of twenty-five mixing units. In contrast, there was no significant difference in the mixing efficiency between the mixers with four sets of twenty-five mixing units and a single set of one hundred mixing units (the detailed geometrical design is shown in Figure 6). The results suggest that the mixing efficiency deteriorated when the orientation of each set of asymmetric obstacle structures was reversed with respect to the flow direction in the microchannel before the mixing process proceeded sufficiently. If the five mixing units for the original set are regarded as a single mixing unit, the geometric features of the mixer can be considered symmetrical with respect to a point on the longitudinal centerline of the microchannel. According to the results shown in Figure 5, the asymmetric orientation of the mixing units should be maintained up to a microchannel length of at least 4 mm, where the mixing proceeds after passing through approximately 20 mixing units. The experimental results suggest that the highly asymmetric geometric features over an appropriate length enhanced mixing.

### 3.4. Design Optimization of the Asymmetric Planar Micromixers

In this section, we experimentally investigated the influence of the geometrical configurations and dimensions of the asymmetric mixer on the mixing efficiency in the Reynolds number (*Re*) range of 0.13 to 13 (equivalent to total flow rates ranging from 1 to 100 µL/min). The detailed geometrical design of the entire asymmetric mixer employed in the experiments is shown in Figure 6, in which the micromixers consisted of a single set of 100 mixing units fabricated in a rectangular microchannel (200 µm in width and 54 µm in height). The mixing efficiencies of six different types of mixing units (M1–M6) were investigated. For comparison, a serpentine micromixer (M7) with a perfectly symmetric mixing unit with respect to the longitudinal centerline of the microchannel was also investigated. Here, the serpentine micromixer was strictly asymmetric with respect to the longitudinal centerline of the microchannel, but a symmetrical geometry can be rendered by moving a set of obstacle structures arranged on one sidewall in the downstream direction. As examples of mixing units, the geometrical configurations of M1, M4, and M7 are illustrated in Figure 6. Here, the total distance connecting the center positions in the width-wise direction of the microchannel in a single mixing unit is defined as the unit flow path length (UL), corresponding to the solid red line in each mixing unit. The geometric dimensions of the obstacle structures (BL1–BL4 and UL) of all mixers are summarized in Table 2. In this study, the asymmetric geometrical feature can be defined as the state in which the obstacle structures arranged on both sidewalls differ from each other in at least one or more of their lengths, the distance between each other along both sidewalls, and the total number on each sidewall.

**Figure 7:**
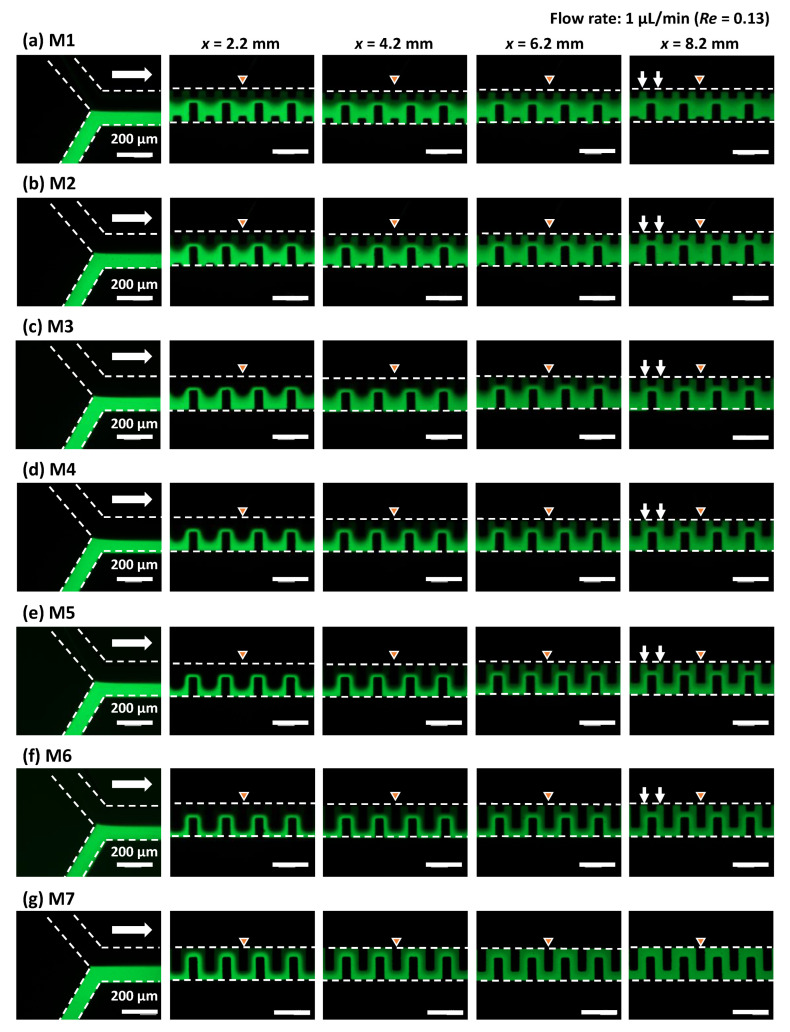
Experimental investigation of the mixing behaviors of six different types of asymmetric micromixers (**a**) M1, (**b**) M2, (**c**) M3, (**d**) M4, (**e**) M5, (**f**) M6, and (**g**) the serpentine micromixer (M7) with a perfectly symmetric mixing unit. Pure water and fluorescein-dyed water (0.1 mol/m^3^) were introduced at the same flow rate of 0.5 µL/min (a total flow rate of 1 µL/min and *Re* = 0.13).

Figure 7 shows the fluorescence microscopy images of the flow behavior of pure water and fluorescein-dyed water (0.1 mol/m^3^) introduced into the microchannel (200 µm in width and 54 µm in height) at each flow rate of 0.5 µL/min, which is equivalent to a total flow rate of 1 µL/min and *Re* = 0.13 in the microchannel. The flow profile in the asymmetric mixers (M1–M6) exhibited laminar flow within a microchannel length of *x* = 4.2 mm (after passing through 20 mixing units). The interface between the two fluids gradually disappeared at *x* = 8.2 mm (40 mixing units), possibly due to the prevailing action of molecular diffusion at an extremely low *Re* = 0.13. However, mixing was less efficient in the narrow spaces along one sidewall of the microchannel, as indicated by the arrow in the photographs. In contrast, the symmetric serpentine micromixer (M7) exhibited superior mixing performance compared with the asymmetric mixers, in which the green fluorescence intensity across the microchannel width was almost uniform at *x* = 8.2 mm (40 mixing units). This could be due to the absence of narrow spaces in the microchannel.

**Figure 8:**
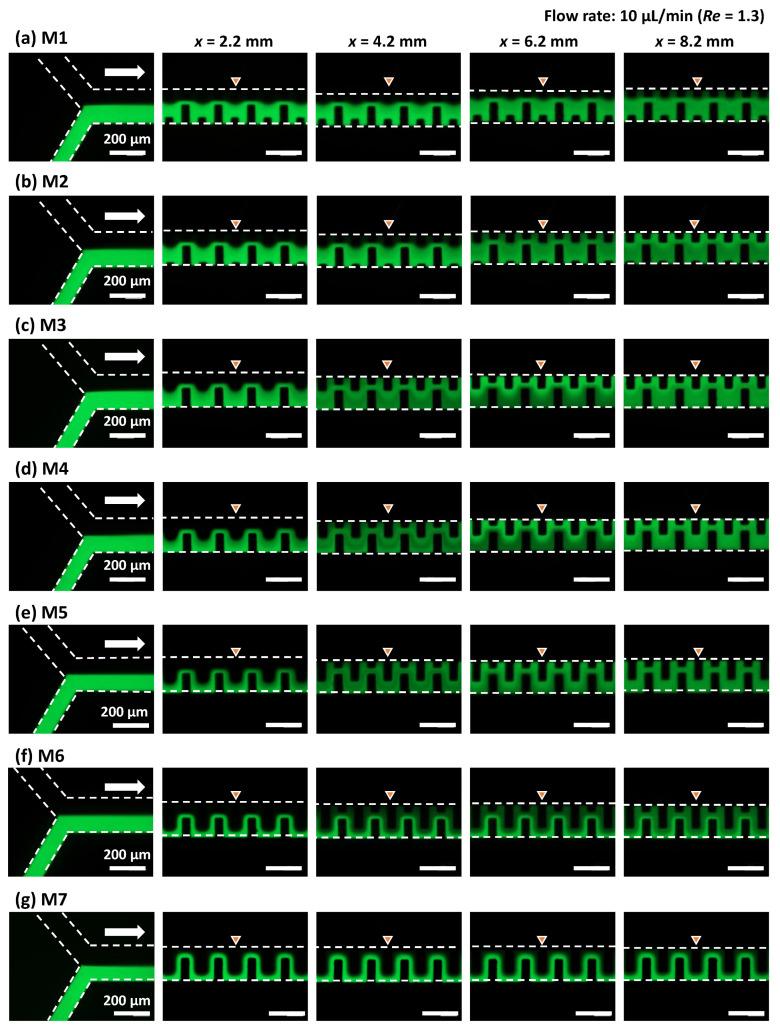
Experimental investigation of the mixing behaviors of (**a**–**f**) six different types of asymmetric micromixers (M1–M6) and (**g**) the serpentine micromixer (M7) with a perfectly symmetric mixing unit. Pure water and fluorescein-dyed water (0.1 mol/m^3^) were introduced at the same flow rate of 5 µL/min (a total flow rate of 10 µL/min and *Re* = 1.3).

Figure 8 shows the mixing behavior at a total flow rate of 10 µL/min (*Re* = 1.3). In the case of mixer M1, fluorescein-dyed water gradually spread across the microchannel width as the mixing proceeded along the microchannel. However, the interface between the two fluids was still clearly visible, even at a microchannel length of *x* = 8.2 mm after passing through 40 mixing units. Compared with mixer M1, the mixing performance of mixer M2 was improved, in which an almost equal distribution of fluorescence could be observed at *x* = 8.2 mm (40 mixing units). However, mixers M3–M5 exhibited different mixing behaviors. It can be seen that an alternative lamellar pattern consisting of fluorescein and water appeared at *x* = 4.2 mm (20 mixing units). Subsequently, the fluorescence intensity on the left-hand side of the microchannel against the flow direction became stronger than that on the right-hand side at *x* = 6.2 mm (30 mixing units). This change in flow position through asymmetric mixing units promoted enhanced mixing, achieving almost uniform mixing at *x* = 8.2 mm (40 mixing units). In contrast, the mixing performance of mixer M6 deteriorated, in which the identifiable interface between the two fluids was still maintained even at *x* = 8.2 mm (40 mixing units). This could be because the geometric features of the mixing units in M6 were asymmetric with respect to the longitudinal centerline of the microchannel but approached a symmetric geometrical design compared with the mixing units M3–M5. This hypothesis is supported by the fact that the serpentine micromixer (M7), with perfectly symmetric mixing units, exhibited the lowest mixing performance.

**Figure 9:**
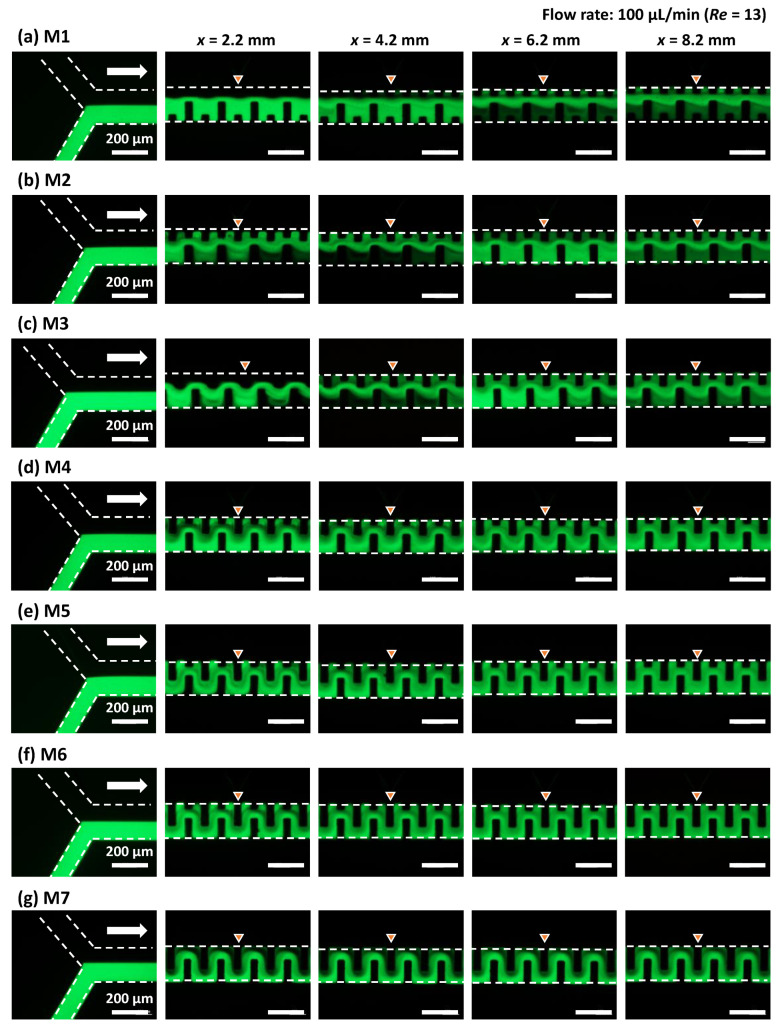
Experimental investigation of the mixing behaviors of (**a**–**f**) six different types of asymmetric micromixers (M1–M6) and (**g**) the serpentine micromixer (M7) with a perfectly symmetric mixing unit. Pure water and fluorescein-dyed water (0.1 mol/m^3^) were introduced at the same flow rate of 50 µL/min (a total flow rate of 100 µL/min and *Re* = 13).

Figure 9 shows the mixing behaviors at a total flow rate of 100 µL/min (*Re* = 13). The flow pattern and mixing behaviors were roughly similar for mixers M1–M3. The mixing proceeded by alternatively changing the flow position of the two fluids. Furthermore, an alternative lamellar pattern appeared even at *x* = 2.2 mm (10 mixing units) in mixers M4–M6. Eventually, there was no identifiable interface between the two fluids at *x* = 6.2 mm (30 mixing units). The different mixing behaviors between M1–M3 and M4–M6 depended on the degree of physical obstruction posed by the obstacle structures with different lengths of BL3 (in the range of 40–140 µm) when the converged (two) fluids flowed out from the narrow gap (40 µm) between BL1 and BL2 (Figure 6). In contrast, the flow profile in the symmetric serpentine micromixer (M7) exhibited laminar flow even at *x* = 8.2 mm (40 mixing units), although the interface between the two fluids gradually disappeared as the mixing proceeded along the microchannel.

**Figure 10:**
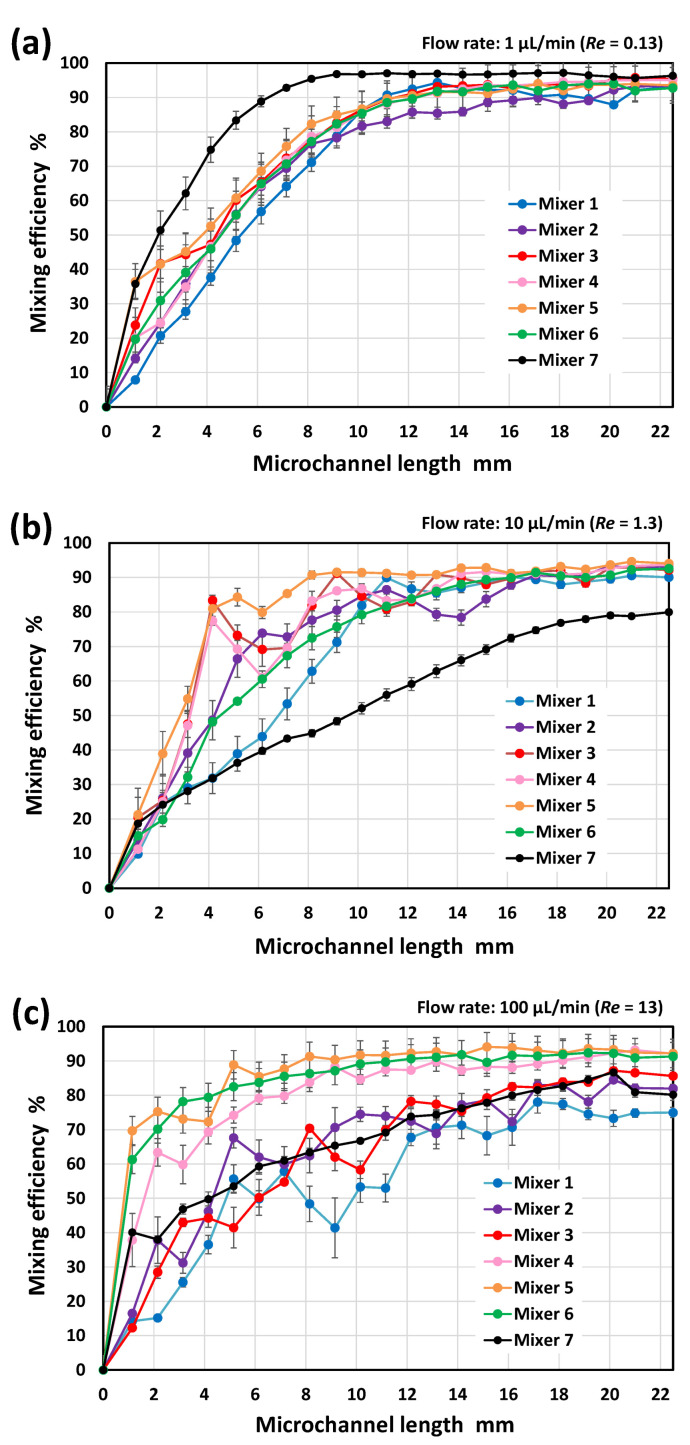
Experimental results showing the mixing efficiencies of six different types of asymmetric micromixers (M1–M6) and the serpentine micromixer (M7) at a total flow rate of (**a**) 1 µL/min and *Re* = 0.13, (**b**) 10 µL/min and *Re* = 1.3, and (**c**) 100 µL/min and *Re* = 13.

Figure 10 shows the resulting mixing efficiencies of the asymmetric mixers (M1–M6) and the symmetric serpentine mixer (M7) as a function of the microchannel length (*x*) at a total flow rate of (a) 1 µL/min and *Re* = 0.13, (b) 10 µL/min and *Re* = 1.3, and (c) 100 µL/min and *Re* = 13; the vertical bars represent the standard deviation for each mixer in the three experiments. As shown in Figure 10a, the mixing rate of mixer M1 was slightly delayed relative to the other asymmetric mixers (M2–M6), possibly due to it having the shortest unit flow path length (UL = 280 µm) (Table 2). However, after passing through 50 mixing units corresponding to a microchannel length of *x* = 10 mm, the mixing efficiencies of all the asymmetric mixers (M1–M6) reached 80% or more. The results show that the geometric features of the asymmetric mixers did not significantly affect the mixing efficiency. At an extremely low Reynolds number (*Re* = 0.13), in which molecular diffusion dominates the mixing process, although the serpentine micromixer (M7) without stagnant regions on the microchannel sidewall (narrow spaces indicated by the arrow in Figure 7) exhibited better mixing performance than the asymmetric mixers, no significant difference in the resulting mixing efficiency was observed. The mixing efficiency was approximately 90% at *x* = 20 mm (100 mixing units).

However, the mixing performance strongly depended on the geometric features of the mixers as the Reynolds number increased to 1.3 (Figure 10b). The efficiency of mixing in the symmetric serpentine micromixer (M7) was significantly reduced compared with that in the asymmetric mixers (M2–M6). Among the asymmetric mixers, mixers M3–M5 achieved a significantly higher mixing efficiency (of approximately 80% at *x* = 4 mm after passing through only 20 mixing units) compared with the other asymmetric mixers (M1, M2, and M6). However, the difference in mixing efficiency decreased as the mixing progressed downstream, eventually reaching approximately 90% at *x* = 20 mm (100 mixing units), whereas the mixing efficiency of the serpentine mixer (M7) was only 80%.

With the further increase in the Reynolds number by 13 (Figure 10c), the mixing performance can be roughly divided into two groups, mixers M1–M3 and M4–M6. These results are consistent with the distinctive features of the mixing processes observed by fluorescence microscopy (Figure 9). Mixers M4–M6 achieved a high mixing efficiency of 80% or more even at *x* = 6 mm (30 mixing units) and subsequently attained approximately 90% mixing efficiency at *x* = 20 mm (100 mixing units), whereas the mixing efficiency of mixers M1–M3 was only 80%. The serpentine mixer (M7), consisting of symmetric mixing units, also exhibited poor mixing performance compared with the asymmetric mixers M4–M6. It should be noted that the asymmetric mixer M6 showed a higher mixing performance than the symmetric serpentine mixer (M7), even though the geometric features of the two mixers were similar. A comparison of the experimental data revealed that the asymmetric mixer M5 demonstrated the best mixing performance over a wide range of Reynolds numbers (0.13 ≤ *Re* ≤ 13), followed by mixer M4, whereas the other asymmetric mixers exhibited suitable mixing performance only in a specific range of Reynolds numbers.

### 3.5. Experimental Investigation of Pressure Drop in the Asymmetric Planar Micromixers

The pressure drops in the asymmetric micromixers (M1–M6) were experimentally investigated and compared with those in the serpentine micromixer (M7) and a rectangular microchannel without mixing units (200 µm in width and 54 µm in height). In the experiments, water (colored with blue food color, 0.1% *w*/*v*) was introduced into a microchannel from one of the two inlet ports (the other was not opened in the PDMS devices). In the experiments, the geometric designs of the mixers were similar to those shown in Figure 6 and Table 2 (a single set of 100 mixing units).

**Figure 11:**
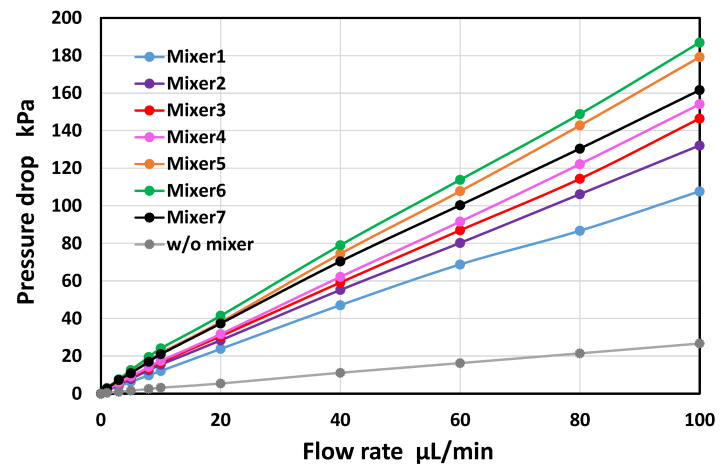
Experimental results showing the pressure drops of six different types of asymmetric micromixers (M1–M6) as a function of the flow rate in the range of 1 to 100 µL/min (*Re* = 0.13–13). For comparison, the data obtained in a rectangular microchannel without mixing units and a serpentine micromixer (M7) are included in the graph.

As shown in Figure 11, the pressure drop increased linearly with the increasing flow rate in all the experiments. The slope (kPa/(µL/min)) of the linear regression line passing through the origin and coefficient of determination (R^2^) are summarized in Table 3. A strong correlation was obtained. For the rectangular microchannel without the mixing units, the pressure drop reached 27 kPa at a flow rate of 100 µL/min. According to the theory of straight rectangular microchannels [44,47], the theoretical pressure drop at 100 µL/min (*Re* = 13) can be calculated to be 26 kPa, in which the total length of the microchannel is 2 mm for a width of 100 µm and 28.87 mm for a width of 200 µm, for a height of 54 µm (Figure 6). The measured value was in good agreement with the theoretical value. The pressure drops of the asymmetric mixers M5 and M6 were greater than those of the serpentine mixer (M7), even though M7 featured the longest unit flow path (Table 2). In contrast, the other asymmetric mixers (M1–M4) exhibited a pressure drop below the value of M7. In terms of the mixing cost (defined as the ratio of mixing efficiency to pressure drop [16]), the asymmetric mixer M4 was the most efficient micromixer over a wide range of Reynolds numbers (0.13 ≤ *Re* ≤ 13) compared with the mixer M5, which had the best mixing efficiency.

### 3.6. Mixing Mechanism of the Asymmetric Planar Micromixers

**Figure 12:**
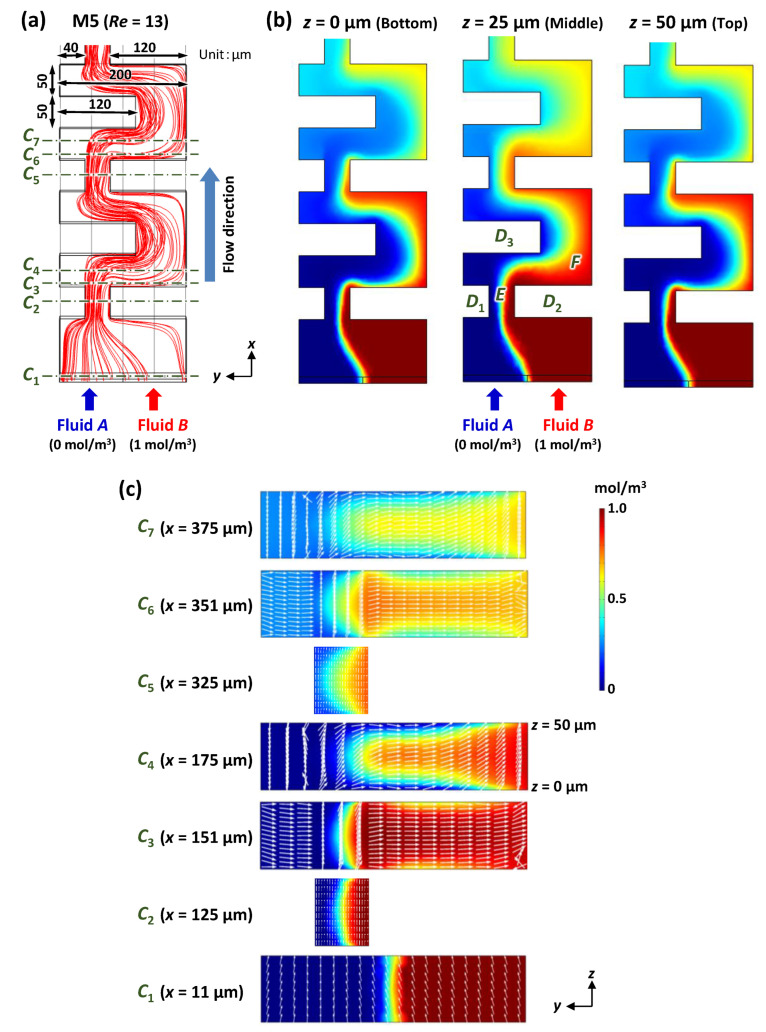
Simulation results showing (**a**) the flow streamlines, (**b**) the concentration contours of two different fluids (with a concentration of 0 and 1 mol/m^3^) on the bottom surface (*z* = 0), middle surface (*z* = 25 µm), and top surface (*z* = 50 µm) of the microchannel in the asymmetric micromixers (M5) at a flow velocity of 8.3 × 10^–2^ m/s each (equivalent to a total flow rate of 100 µL/min and *Re* = 13), and (**c**) the concentration distribution (contour) and transverse velocity fields (arrows) across the microchannel cross-section at different *x* positions (*x* = 11–375 µm).

In this section, we discuss the mixing mechanism of the asymmetric micromixers. Figure 12 shows the computational analysis of the mixing phenomena in the asymmetric micromixers (M5). Blue-colored fluid *A* (0 mol/m^3^) and red-colored fluid *B* (1 mol/m^3^) were introduced into a microchannel (200 µm in width, 50 µm in height, and 1350 µm in length; FEM model) at a flow velocity of 8.3 × 10^–2^ m/s each, equivalent to 50 µL/min. The total flow rate and Reynolds number (*Re*) were 100 µL/min and 13, respectively. First, fluids *A* and *B* were contracted by two obstacle structures (denoted by *D*_1_ and *D*_2_) at an asymmetric position with respect to the longitudinal centerline of the microchannel. After flowing out from a narrow gap (denoted by *E*) located on the left-hand side of the microchannel, fluids *A* and *B* rapidly expanded over a large area (denoted by *F*) by changing their flow directions from left to right across the longitudinal centerline of the microchannel, to bypass an obstacle structure (denoted by *D*_3_). At this moment, fluid *B* could easily expand into the large area *F* and was then most likely to occupy room *F* owing to a sudden decrease in the velocity of the flowing fluid *B*, whereas the expansion of fluid *A* was difficult because of insufficient space to flow into. As shown in Figure 12c, fluid *B* dominated the central portion of the microchannel in the height direction (at approximately *z* = 25 µm), whereas fluid *A* flowed upward (toward the top surface at *z* = 50 µm) and downward (toward the bottom surface at *z* = 0) to avoid fluid *B*. This could be attributed to the fact that the flow velocity decreased toward the top and bottom surfaces in the microchannel according to the parabolic velocity profile in pressure-driven fluid flow; that is, fluid *A* had a higher possibility of flowing into spaces with lower velocities than fluid *B*. It can be concluded that this flow behavior increased the interfacial contact area between the two fluids, thus promoting effective mixing in asymmetric mixers. According to the mixing mechanism of the asymmetric mixers proposed here, we term it a P-ACE micromixer.

**Figure 13:**
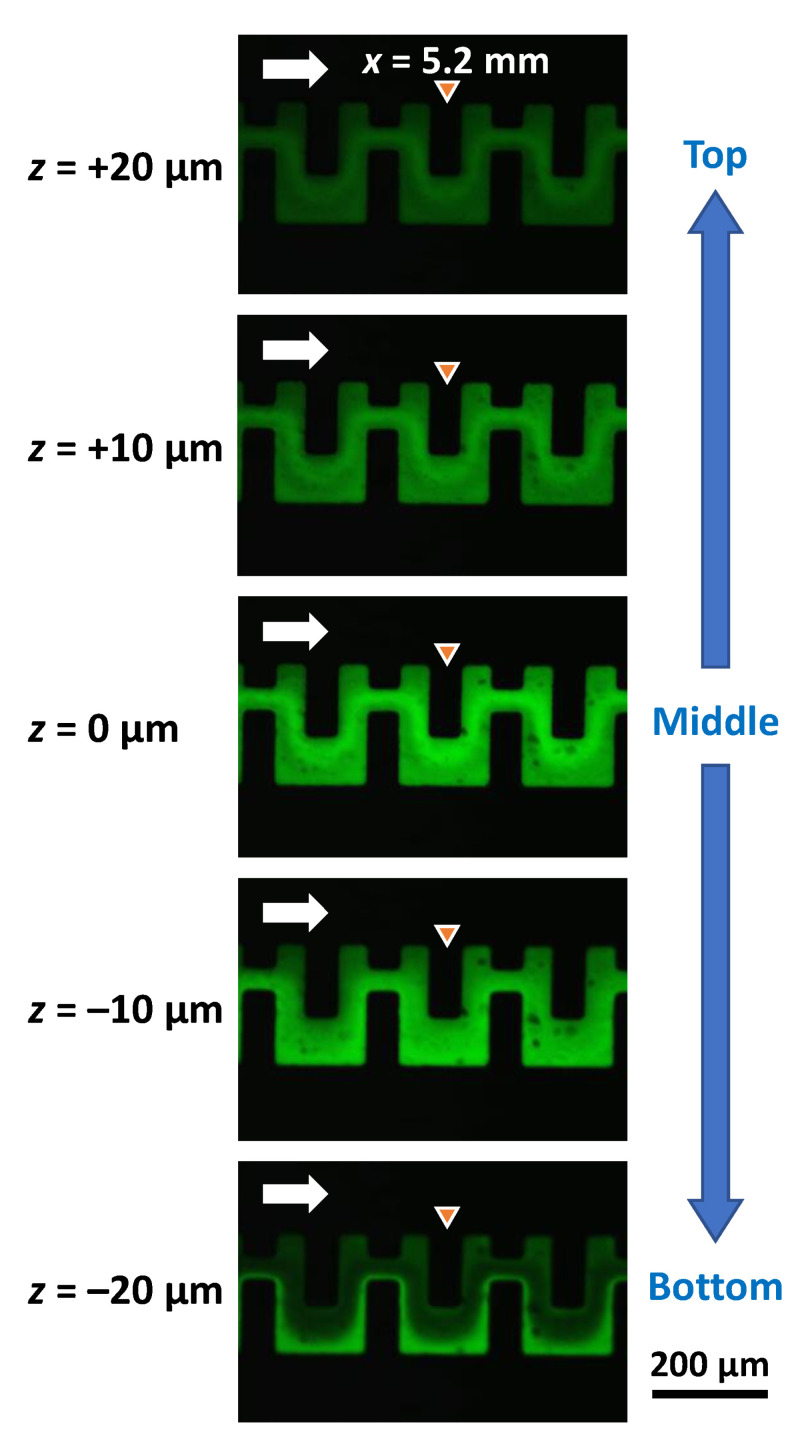
Confocal microscopy images of the mixing behavior at different focal *xy*-planes with a distance of 10 µm in the height direction (*z*-axis direction), in which *z* = 0 is the middle plane of the microchannel height, and *z* = –20 µm and *z* = 20 µm are the top and bottom surfaces of the microchannel, respectively. Pure water and fluorescein-dyed water (0.1 mol/m^3^) were introduced into the asymmetric micromixers (M5) at each flow rate of 5 µL/min, equivalent to a total flow rate of 10 µL/min and *Re* = 1.3 in the microchannel.

Figure 13 shows the confocal microscopy images of the mixing behavior in the asymmetric micromixers (M5) at each flow rate of 5 µL/min for pure water and fluorescein-dyed water (0.1 mol/m^3^), equivalent to a total flow rate of 10 µL/min and *Re* = 1.3 in the microchannel (200 µm in width and 54 µm in height). Confocal microscopy imaging was performed using a multiphoton confocal microscope (A1R MP, Nikon, Tokyo, Japan). The five images (at approximately *x* = 5.2 mm after passing through 25 mixing units) were acquired at different focal *xy*-planes with a distance of 10 µm in the height direction (*z*-axis direction) of the microchannel, in which *z* = 0 is the middle plane, and *z* = –20 µm and *z* = +20 µm are near the bottom surface (PDMS; water contact angle *θ* = 108°) and the top surface (silicone-based adhesive tape; *θ* = 102°) of the microchannel, respectively. The relative fluorescence intensity of the image acquired at *z* = 0 was significantly higher than those of the images acquired at *z* = –20 µm and *z* = +20 µm. This could be attributed to the fact that pure water was flowing up and down toward the top and bottom surfaces, respectively, to avoid the fluorescein-dyed water. The distinctive feature of the mixing process was in good agreement with the simulation results (Figure 12). It can also be seen that the fluorescence intensity along the right-hand sidewall of the microchannel against the flow direction was stronger than that in the other regions at *z* = –20 µm, whereas the flow directions of the two fluids changed for z > 0 µm, resulting in higher fluorescence intensity along the left-hand sidewall of the microchannel. The results revealed that the secondary flow was generated in the cross-sectional *yz*-plane of the microchannel by the asymmetric obstacle structures.

It can be concluded that the highly asymmetric geometric features of the P-ACE micromixers developed in this study played a crucial role in promoting enhanced mixing over a wide range of Reynolds numbers (0.13 ≤ *Re* ≤ 13). As described in the introduction, planar micromixers generally exhibit the lowest mixing performance at *Re* ≈ 1–10 [16,24,36,37,39]. There are two main reasons behind this. The first reason is that the effect of molecular diffusion becomes smaller than that in the lower Reynolds number regime, owing to a reduction in the residence time with an increase in *Re*. Second, the Dean vortices induced by centrifugal forces cannot be expected to promote enhanced mixing compared with the mixing process in a higher Reynolds number regime. To the best of our knowledge, planar micromixers with mixing units shaped as interlocking-semicircle (ILSC) and omega (Ω) channel designs exhibit superior mixing performance compared with other reported planar micromixers, achieving a mixing efficiency of 80% or more for a wide range of Reynolds numbers (*Re* = 0.01–50) [48]. However, a specifically designed mixing module (a series of ILSC or Ω designs) must be integrated into microfluidic devices. In contrast, the P-ACE micromixers proposed here can not only be easily incorporated into a part of a microchannel network without compromising the original design of microfluidic devices but can also be fabricated by only a single-step photolithography process. To provide the design guidelines for optimizing the P-ACE micromixer, we will further investigate the effect of the geometrical parameters, for example, the width and height of a microchannel, and the pitch of asymmetric obstacle structures, on the mixing performance.

## 4. Conclusions

We introduced a simple and efficient planar passive micromixer with asymmetric vertical obstacle structures with respect to the longitudinal centerline of the microchannel, termed a P-ACE micromixer, which can be easily fabricated via only a single-step photolithography process and integrated into a microfluidic device without changing the original device design. The mixing process begins with the contraction of two fluids to an asymmetrical position near one sidewall with respect to the longitudinal centerline of the microchannel, followed by the abrupt expansion of the two fluids across the centerline into an asymmetrically large space on the other side. By repeating this procedure, the interfacial contact area between the two fluids increases, thus promoting mixing. As a result, a high mixing efficiency of the optimized P-ACE micromixer could be achieved over a wide range of the Reynolds number (0.13 ≤ *Re* ≤ 13)—a mixing efficiency of 80% or more at a microchannel length of 10 mm, and eventually attaining approximately 90% mixing efficiency within 20 mm. We will explore a more appropriate asymmetric geometrical feature for further improvement in the mixing performance of the P-ACE micromixer. In future studies, the P-ACE micromixer developed here will be integrated into microfluidic diagnostic devices to provide a rapid and easy sample-to-answer platform for multiplexed genetic diagnosis of multiple nucleic acid (DNA/RNA) targets with a more simplified operating procedure for our targeted applications [42,43,44,45].

## Figures and Tables

**Table 2:** Geometric dimensions of the obstacle structures of six different types of mixing units integrated into the asymmetric micromixers (M1–M6) and a serpentine micromixer (M7) with a symmetric mixing unit (Figure 6).

Mixer	BL1 (µm)	BL2 (µm)	BL3 (µm)	BL4 (µm)	UL (µm)
M1	40	120	40	40	280
M2	40	120	60	20	320
M3	40	120	80	0	360
M4	40	120	100	0	380
M5	40	120	120	0	400
M6	40	120	140	0	420
M7	0	140	140	0	480

**Table 3:** Relationship between pressure drop in the micromixers and flow rate in the range of 1 to 100 µL/min (*Re* = 0.13–13). The slope of the linear regression line passing through the origin and the coefficient of determination (R^2^) are summarized.

Mixer	Slope (kPa/(µL/min))	R^2^
M1	1.10	0.9986
M2	1.33	0.9997
M3	1.46	0.9996
M4	1.54	0.9998
M5	1.80	0.9996
M6	1.89	0.9992
M7	1.65	0.9983
w/o	0.27	0.9996

## Data Availability

The data presented in this study are openly available online at doi:10.3390/mi13091386.
